# Neighbourhood environment and depressive symptoms among the elderly in Hong Kong and Singapore

**DOI:** 10.1186/s12942-020-00238-w

**Published:** 2020-11-13

**Authors:** Winnie W. Y. Lam, Becky P. Y. Loo, Rathi Mahendran

**Affiliations:** 1 Department of Geography, The University of Hong Kong, Pokfulam Road, Hong Kong, China; 2 Department of Psychological Medicine, National University of Singapore, Singapore, Singapore

**Keywords:** Depressive symptoms, Elderly, Neighbourhood environment, Hong kong, Singapore

## Abstract

**Background:**

Geriatric depression is a growing public health issue worldwide. This study aims at identifying the relevant neighbourhood attributes, separate from the individual-level characteristics, that are related to the onset of depressive disorders among the geriatric population.

**Methods:**

This study adopts a structural equation modelling (SEM) approach to understand the effect of the neighbourhood environment on geriatric depression, as identified by data collected from community-dwelling elderly living in Hong Kong and Singapore. Using network buffers as the unit of analysis, different features of the neighbourhood environment are captured and analysed. SEM also examines the strength and direction of the relationships using different parameters at both the individual and neighbourhood levels, as well as the prevalence of depressive symptoms among the elderly.

**Results:**

The total sample size is 347, with 173 and 174 elderly people in Hong Kong and Singapore respectively. The results show that in addition to one’s physical health status, both objective and subjective neighbourhood factors including the size of parks, land use mix, walkability, and connectivity are all statistically significant influential factors in geriatric depression. In particular, enhancing walkability and providing more parks at the neighbourhood level can bring mental health benefits.

**Conclusions:**

Public health policy initiatives aimed at tackling geriatric depression can be achieved by adopting a holistic and integrative approach to better prepare the neighbourhood environment in an ageing society.

## Introduction

Depression in later life is an important public health issue due to the increased risk of morbidity and suicide, as well as decreased physical, social, and cognitive functioning [5]. In two of the most rapidly ageing cities in Asia, elderly depression is one of the most common psychiatric disorders. In Hong Kong, the prevalence rate is 13.7% for females and 8.9% for males; while in Singapore it is estimated at 5.5% [20, 44]. Therefore, to be effective and relevant, age-related policies and programmes in Asian cities need to keep pace with the rapidly ageing population [37]. In particular, the identification of factors that exacerbate or alleviate depression have important implications in promoting healthy ageing-in-place, a concept that allows the elderly to live in their existing communities with familiar family and/or social support, rather than to move to institutionalized homes for the elderly [32].

In cities, multiple factors contribute to the risk of depression ranging from genetic vulnerabilities, neurobiological changes, and environmental factors such as social and psychological issues, and often the neighbourhood environment [47]. For governments, a holistic ageing policy that considers not only individual demographic, medical, and socio-economic factors but also their neighbourhood environment is needed to safeguard the physical and mental health of the elderly. In particular, the integrative conceptual framework developed by Billings and Moos [4] suggests that personal resources interact with environmental resources such as physical and architectural features of community settings which could lead to environmental stressors and affect appraisal and coping responses to cause depression. This research focusing on the neighbourhood environment and elderly depressive symptoms is grounded in this theoretical background.

**Fig. 1:**
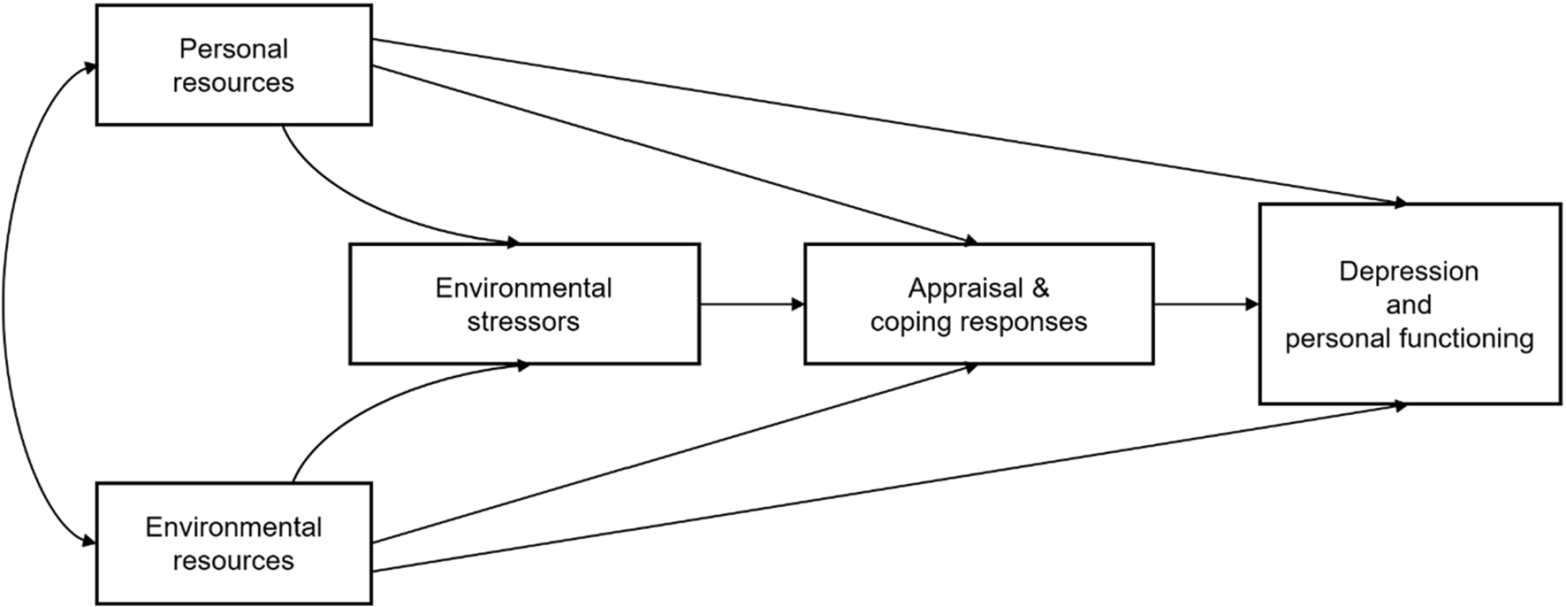
An integrative framework of people-environment factors in analysing adaptive processes and depression [4]

Figure 1 is a schematic diagram of the integrative framework. A recent review summarises that neighbourhood environmental resources including high walkability, connectivity, and accessibility are expected to contribute to the elderly’s physical and mental health, and hence their quality of life [33]. The elderly tend to spend more time in their neighbourhood environment because their activities are significantly curtailed by physical decline and frailty, retirement, reduced mobility, decreased access to transport, shrinking social support and networks, and cognitive decline [12]. Hence, satisfaction with the availability and proximity of key resources, such as retail and healthcare services, is also found to reduce depressive symptoms [24]. Good connectivity, while reducing trip distance between locations, enhances walkability and encourages outdoor mobility in older adults [42]. Similarly, open spaces can improve living conditions and enhance the overall quality of life [23]. A geographical neighbourhood which is not supportive to walking, and with limited access to services and resources, poses further significant barriers for older people seeking full participation in society, with deleterious effects predisposing the development of geriatric depressive symptoms [13, 41]. Moreover, several studies have shown that certain neighbourhood factors were consistently associated with depression [14, 35, 51, 52]. Indeed, it is found that seniors facing severe mobility impairment have a lower level of social engagement and life satisfaction [29], suggesting that poor accessibility can create environmental stressors to the elderly.

In terms of the interactions of personal and environmental resources, the prevalence of depression has been found to be affected by the number of rooms in the home, as well as housing quality [14]. A higher satisfaction with housing was associated with fewer depressive symptoms [28]. Several self-reported measures of the geographical neighbourhood, such as neighbourhood collective efficacy, neighbourhood problems, and neighbourhood quality, were also important. Generally, living in a low-income neighbourhood appears to be strongly related to poorer mental health and depression [16]. The extent, strength, and quality of social connections with each other in their neighbourhoods affect their appraisal and coping responses and, hence, is also a social determinant of health [19]. ‘Non-material’ aspects of life, such as support and love, are necessary for good mental health [3]. Hence, the emotional support older people receive within their local neighbourhood is particularly relevant. Studies suggest that the concentration of older people living close together could have a favourable effect on alleviating geriatric depression [35]. There is also a body of evidence showing that social capital—the degree to which older people see themselves as involved with, and are able to rely on, other members in their neighbourhood [40]—buffers against stressful life events, improves coping responses, and helps prevent the onset of depression [26]. Social capital has important moderating or mediating effects on depression in different population-based cohort studies [3], including a sampled Chinese population [6].

Despite the growing interest in understanding the environmental influences on people’s health, research studies have mainly focused on the West. Much is still unknown about the importance of the neighbourhood environment on depression in Asian communities, which tend to be of higher density and diversity [9, 51, 52]. The study by Chen et al. focused on the neighbourhood characteristics of elderly people living in low-income communities in Hong Kong. By assessing perceived measures using linear regression, they found that at-ease walking proximity to medical facilities was influential [9]. The work by Chen’s team adds to the knowledge about particular aspects of neighbourhood environmental resources on geriatric mental health in Asian communities. Moreover, the empirical evidence in three Asian cities (i.e., Hong Kong, Singapore, and Tokyo) indicates that the home environment of the elderly, especially neighbourhood walkability, is interconnected with the formation of social capital and the elderly’s physical and mental health conditions [34]. The research team of Zhang et al. [51, 52] further explored the environment-depressive symptoms associations and found that the “ultra-dense, well-connected, pedestrian-friendly, destination-rich neighbourhoods may contribute to lowering the risk of depressive symptoms in Hong Kong older adults by enabling them to frequently walk to local destinations of daily living and, thus, maintain their independence and bond with the community” (Zhang et al. [52]: 96). Yet, exposure to extreme levels of public transport density and associated traffic volumes may create environmental stress and increase the risk of depressive symptoms [51].

Our study aims to complement and expand the existing literature by revisiting the definition of geographical neighbourhood, incorporating both objective and subjective measures of the neighbourhood, and by extending the study area to include both Hong Kong and Singapore—two cosmopolitan Asian cities—to elucidate effective strategies ahead. It is increasingly recognized that a combination of objective and subjective measures is necessary in understanding complex public health issues [22]. By employing structural equation modelling (SEM), we examine the possible mechanisms by which depressive symptoms may be related to the neighbourhood environment in which a person resides. Hong Kong and Singapore were selected in view of their many common characteristics, physically, economically, historically, and culturally. The contrast between the two societies may well serve to inform the debate on geriatric depression, which thus far has centred primarily on Western countries.

## Methodology

### Sample population and study area

This cross-sectional study involved community-dwelling senior citizens in Hong Kong and Singapore with support from local senior citizen community centres. Due to the lack of comprehensive sampling frames, convenience sampling was used. The survey altogether included 228 seniors in Hong Kong and 250 seniors in Singapore. Written consent was obtained from all participants before the interviews, which were conducted during May and June, 2013. More details about the survey design and sample recruitment are provided in Loo et al. [33]. The neighbourhoods in Hong Kong were selected based on the comprehensive list of senior community centres from the Social Welfare Department. All senior community centres were approached for participation. Upon initial agreement to participate, site visits were done to establish the suitability of the venue, time schedule, and membership size. The samples eventually came from four neighbourhoods in Sai Ying Pung on Hong Kong island, Hung Hom and Lai Chi Kok in Kowloon, and Tai Po in the New Territories. In Singapore, the seniors were recruited at an established training and research centre in the Jurong town area. This is the same large scale satellite housing development that is seen across the country; each is self-contained with public housing units, a town centre, amenities for shopping, employment in industrial estates, schools, medical care and recreational facilities. Based on the respondents’ home locations, the three neighbourhoods are Jurong West Central, Boon Lay, and Hong Kah. Major road networks generally do not run through these townships which are however linked to these major highways. In both cities, our target population were healthy community-dwelling elderly who are not experiencing any acute life event known to the elderly community or research training staff.

Based on the samples, further inclusion criteria for the study population were people who (a) were aged 65 years or older in the study period; (b) scored 20/22 or above in MMSE during the screening of cognitive abilities (see below); and (c) completeness of the questionnaires. This resulted in a total sample size of 347, with 173 and 174 elderly people in Hong Kong and Singapore respectively. More specifically, 26, 47, and 58 samples were excluded because of the above three inclusion criteria, respectively.

### Demographic data collection

The gathered demographic and socioeconomic information of the respondents included: gender, age, educational level, living arrangements and accommodation, household car-ownership, and number of chronic diseases. The body mass index (BMI) for all respondents—as an indicator of general obesity—was also recorded.

### Assessments

The Mini-Mental State Examination (MMSE) was used to measure cognitive impairment. This 30-point questionnaire tests various cognitive functions, including orientation to time and place, repetition, verbal recall, attention and calculation, language, and visual construction. The total test score ranges from 0 (impaired) to 30 (normal). Cut-off points for cognitive impairment of 22 for those who are literate and 20 for those who are illiterate were based on a previously validated study in the Chinese population which yielded a sensitivity of 83.87% and a specificity of 84.48% [49].

The 15-item Geriatric Depression Scale (GDS-15) was used to assess depression. Each item was scored on a dichotomous ‘Yes’ or ‘No’ response, and the scale was the sum of the recoded 15 items, ranging from 0 to 15. Higher scores on the GDS indicate higher levels of depressive symptoms. The prevalence of depression was indicated by a cut-off point of 8 [11]. For Cantonese speakers, the validated Chinese version of the GDS was used. Cronbach’s alpha in the present sample is 0.83, suggesting a reasonably reliable measure for further analysis.

Health-related variables were obtained using the SF-36v2 Health Survey—a multipurpose, short-form health survey. It has 36 questions that yield an eight-scale profile of functional health and well-being, two psychometrically based physical and mental health summary measures, and a preference-based health utility index. The physical component score (PSC) and mental component score (MCS) are two variables used to reflect the general physical and mental health status respectively [33]. Both have a range of 0 to 100 with higher scores suggesting better health [46]. For easy reference, they can be interpreted as summary physical and mental health scores, respectively.

The International Physical Activity Questionnaire—Short Form (IPAQ-SF) was used, which measures health-related participation in physical activity (PA) in populations. The specific types of activity assessed are: walking, moderate-intensity activities, and vigorous intensity activities. Frequency (measured in days per week) and duration (time per day) are collected separately for each specific type of activity. Items are structured to provide separate scores for walking, moderate-intensity, and vigorous-intensity activity, as well as a combined total score to describe overall level of activity. Volume of activity is computed by weighting each type of activity by its energy requirements as defined in the Metabolic Equivalent of Tasks (METs), which are multiples of the resting metabolic rate. Multiplying the MET score with the number of minutes the activity is performed yields a score in MET-minutes. MET-minutes per week were used to classify older people into three categories: low, moderate, and high level of physical activity [21].

### Geographical neighbourhood variables

Indicators of two sets of geographical neighbourhood variables, based on immediacy and breadth of impact, were constructed. Level-1 local factors or individual-specific immediate neighbourhood factors refer to parameters at a micro scale of influence, while those at a meso-level are termed level-2 local factors or wider shared neighbourhood factors. This study advocates the concept of people-based neighbourhoods, which are identified based on the actual activity space or space–time movements of individuals [33] rather than district-based neighbourhoods, which are fixed for administrative purposes. To achieve this, the home addresses of all participants were geocoded using a geographic information system (GIS). To analyse level-1 local factors, a 500-m network buffer was drawn around each participant’s residential location using network distance to reflect walkable distance for the participants [7]. Neighbourhood factors were gaugfed on a pro-rata basis.

The first dimension of level-1 local factors captures the spatial distribution of opportunities near their homes as this plays a key role in defining social in/exclusion [41]. By calculating the shortest physical distance to key facilities around the participants’ homes [50], this study considers the network distance to places such as the nearest medical facility (DIS1) or entrance to an open space (DIS2). Network distance here refers to the length of walking path on the pedestrian network, which is more realistic than the shortest direct line/Euclidian distance, between two places [32]. The second dimension of level-1 local factors captures various key principles in urban planning, including connectivity (CON) and the 3D, that is, density (DEN), diversity (DIV), and design (DES) [8]. CON is measured by the road junction density within the buffer area. For DEN, this study considers overall and elderly population density, where higher density implies an advantage of having more population to support a vibrant neighbourhood. DIV, refers to the mix of land use in contributing to a balanced development. It is calculated using the Simpson’s Diversity Index [43] as an entropy measure by considering five categories of land use: residential, commercial, institutional, recreational, and others [32]. Finally, good DES relates to the attractiveness of the neighbourhood environment. It includes connectivity as expressed in the number of intersections per square kilometre (DES1), the percentage of open space in one’s neighbourhood (DES2a), and the area (km^2^) of parks contained within the network buffer.

The third dimension relates to one’s perception of how walkable is the neighbourhood. Eighteen subjective walkability variables under the dimensions of comfort, convenience and safety were gathered from the face-to-face questionnaire survey. These variables are summarized in Table 1. The different parameters of the neighbourhood walking environment were rated on a Likert scale from one to five, ranging from very poor to very good condition. In addition, participants were also asked to rate the overall walkability of their neighbourhood subjectively on a scale of 0 to 100.

**Table 1:** Subjective ratings of the walking environment in a neighbourhood

Dimension	Variables
Comfort	There are too many people on the streets and it makes it difficult or unpleasant to walk There are covered sidewalks and/or indoor area (e.g. shopping centre) to walk The degree of air pollution is high in my neighbourhood There are enough beautification and enhanced greenery along the streets in my neighbourhood There are many attractive sceneries to look at while walking in my neighbourhood Streets are not clean in my neighbourhood
Convenience	It is easy to walk to transport stations from my home Stores are within easy walking distance of my home There are many alternative routes for getting from place to place in my neighbourhood There are clear road signs and pedestrian signals There are enough facilities to take rest (e.g. benches) Pedestrian bridges or subways are well equipped with sufficient lifts or escalators
Safety	There is so much traffic that it makes me feel insecure to walk on the streets The pedestrian green light time is too short for me to cross the road safely The crime condition in my neighbourhood makes it unsafe to walk Sidewalks conditions are poor (Not flat or slippery) There are many slopes or stairs that make me feel insecure to walk My neighbourhood streets are not well lit at night

To measure social capital, four statements were used. Three statements required a response on a five-point Likert scale from strongly disagree to strongly agree: “People in my neighbourhood get along with each other well”; “People are willing to help each other”; and “Living in this neighbourhood gives me a sense of community”. The fourth statement, “Do you have someone to accompany you outside?”, required a yes or no answer.

The above neighbourhood factors are individual-specific. This poses a challenge to the operationalization of the shared space, which can enhance the relationship between people, places, and the surrounding traffic [17, 18]. Spatial aggregation was then performed in the study by overlapping individual neighbourhood buffers with common boundaries and within a walkable distance roughly double the immediate neighbourhood buffer of 500 m (that is, a radius of roughly 1 km). Level-2 local factors or the wider shared community factors capture the general or average condition of people living there. The first dimension of level-2 local factors captures the average or mean value of all parameters included in the level-1 local factors. The second dimension covers some additional demographic variables. These include the percentage of oldest-old of 85 years or above and females. The third dimension includes an objective walkability assessment, which is a composite score of the walking conditions of the neighbourhood based on the research protocols developed by Loo and Lam [32]. There are altogether twelve variables, covering the pavement (pavement surface, shelter, pedestrian guardrail, directional signs, road works and street furniture) and crossing facilities (dropped kerb, audible pedestrian signals, number of vehicular lanes, refuge island for wide roads, traffic light cycle, and crossing time).

### Research framework

SEM is used to establish relationship strength and direction among the different individual and neighbourhood environment parameters in the occurrence of depressive symptoms among the elderly. It is also used to test the mediating relationship, elucidating the potential pathways in which neighbourhood factors and individual attributes affect depression in the elderly. SEM is particularly suitable to this research because it allows for the building of complex models (including relationships among observed and latent-variables) and corrects for measurement errors, thereby allowing for a more accurate test of the mediational effect [25]. Based on our theoretical framework, we have selected different factors related to personal resources and environmental resources as exogenous variables The latter are further classified as individual-specific immediate neighbourhood characteristics (hereafter referred to as level 1 local factors) and wider shared neighbourhood characteristics (hereafter referred to as level 2 local factors). Depressive symptoms were treated as endogenous. Thus, it is hypothesized that the exogenous variables interact with and mediate the development of depressive symptoms. The dependent variable in this study is the occurrence of depressive symptoms among the elderly population.

## Results

The characteristics of the sample population are shown in Table 2. The overall prevalence of depression using the GDS cut-off point of 8 was 7.8% in Hong Kong and 15.6% in Singapore. It should be noted that convenience sampling was adopted in this research work. In all of the 347 older people, a large proportion (77.2%) were female (*n* = 268). The higher percentage of females in the sample reflects a gender difference in attitudes towards involvement in community and volunteer activities in the two cities [38]. Despite this, the present multi-centre study is an effective method of recruiting sufficient number of community-based subjects. The mean age of the elderly respondents was 73.82 years (SD = 6.04), ranging from 65 to 95 years. 4.9% of the samples belong to the oldest-old age, who were aged 85 or above during the study year. Around 23.3% (*n* = 81) of respondents lived alone and 77.5% were part of non-car owning households (*n* = 269). About 70% of the respondents (*n* = 243) only received primary education or lower level. Following the guidelines of the World Health Organization [48], around two third of respondents were normal-weight individuals (a BMI of 18.5 to 24.99). Nevertheless, 32.6% of the elderly people in this study were overweight and 4.9% were obese. Approximately 23.6% had more than three chronic diseases, of which the top three were high blood pressure (51.8%), knee osteoarthritis (31.1%), and diabetes (18.6%). Despite these constraints, many respondents in Hong Kong remained physically active (45.1%) based on the score in MET-minutes. The share of being physically active was lower in Singapore (6.9%). Moreover, as seen from Table 2, our subject individuals generally enjoyed reasonably good physical and mental health as community-dwelling elderly of over 65 years old. Overall, less than 20% of the respondents have their physical and mental health scores below 50. However, as shown above, these average figures hide problems of depression in the ageing population.

**Table 2:** Descriptive analysis of the sample

Parameters	Hong Kong		Singapore		Total	
	N	%	N	%	N	%
Gender						
Male	20	11.6	59	33.9	79	22.8
Female	153	88.4	115	66.1	268	77.2
Age group						
65–70	30	17.3	87	50.0	117	33.7
71–75	41	23.7	56	32.2	97	28.0
76–80	62	35.8	26	14.9	88	25.4
81–85	27	15.6	3	1.7	30	8.6
Above 85	13	7.5	2	1.1	15	4.3
Living alone	65	37.6	16	9.2	81	23.3
Car-owning household	9	5.2	69	39.7	78	22.5
Education level						
Primary or lower	136	78.6	107	61.5	243	70.0
Junior secondary	20	11.6	34	19.5	54	15.6
Senior secondary or above	17	9.8	33	19.0	50	14.4
BMI group						
Underweight	6	3.5	10	5.7	16	4.6
Normal range	93	53.8	108	62.1	201	57.9
Overweight	65	37.6	48	27.6	113	32.6
Obese	9	5.2	8	4.6	17	4.9
Lifestyle						
Inactive	5	2.9	70	40.2	75	21.6
Minimally active	90	52.0	92	52.9	182	52.4
Active	78	45.1	12	6.9	90	25.9
Three or more kinds of chronic illnesses	42	24.3	40	23.0	82	23.6
Physical health score						
Below 50	36	20.8	9	5.2	45	13.0
50–74	66	38.2	87	50.6	153	44.3
75 or above	71	41.0	76	44.2	147	42.6
Mental health score						
Below 50	13	7.5	8	4.6	21	6.1
50–74	49	28.3	61	35.3	110	31.8
75 or above	111	32.2	104	60.1	215	62.1

**Fig. 2:**
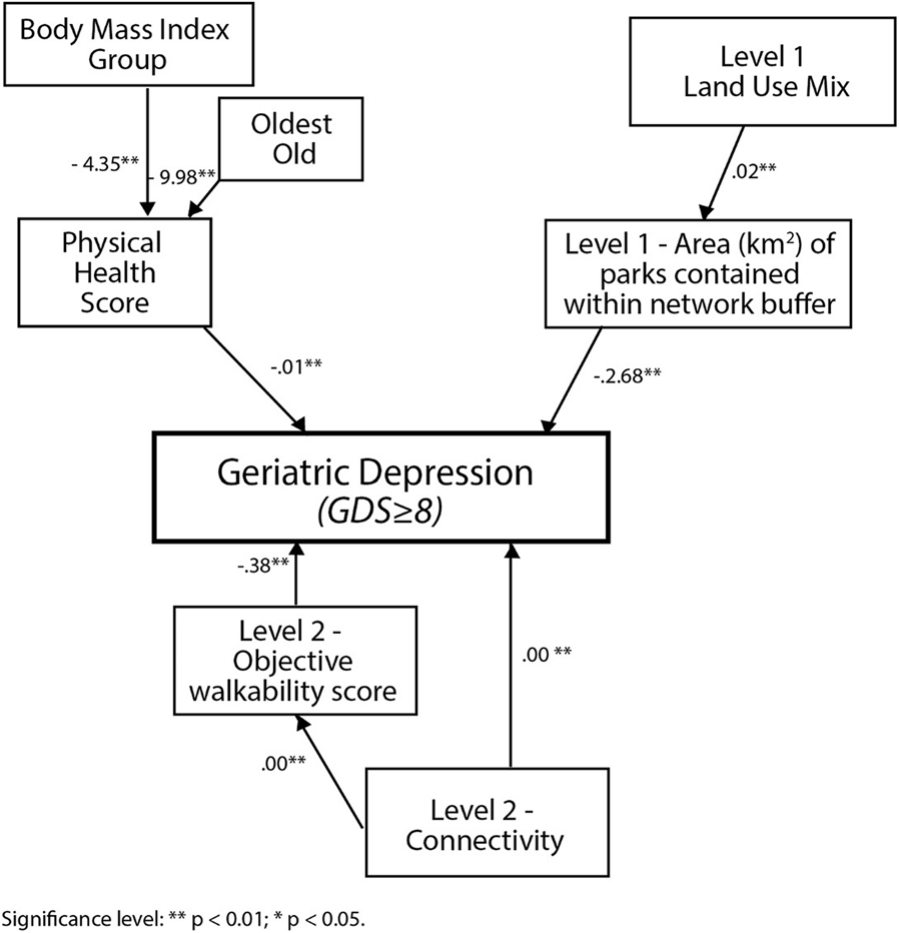
Path diagram showing the relationships between individual and neighbourhood factors

Following the SEM approach, we have tested different models and the final model with accompanying path coefficients is presented in Fig. 2. Only statistically significant variables are kept. Examining the factors that characterize older people’s neighbourhoods may help provide evidence as to the extent to which neighbourhood factors are related to the development of depressive symptoms. Firstly, the model shows that better physical conditions, as reflected by a higher physical health score, are linked to a lower risk in depressive symptoms (coefficient = − 0.01, p < 0.05). While overweight or obese elderly people (coefficient = − 4.35; p < 0.05), and the oldest-old (coefficient = − 9.98; p < 0.05), are less likely to maintain a good physical health status. Among the immediate neighbourhood factors (Level 1), abundant park area (coefficient = − 2.68; p < 0.05) help to counteract depression. The park area is, in turn, affected by land use mix (coefficient = 0.02; p < 0.05), suggesting that higher land use mix is positively associated with the park area. Among the wider shared neighbourhood factors (Level 2), good objective walkability score (coefficient = − 0.38; p < 0.05) is negatively associated with depressive. In addition, the good objective walkability score is positively associated with connectivity (coefficient = − 0.00; p < 0.05), though the variable itself is also directly associated with geriatric depression (coefficient = − 0.00; p < 0.05). Generally, this suggests and reconfirms that higher road junction density (CON or connectivity) can create smaller street blocks to encourage walking but heavy vehicular traffic may also create environmental stress.

In addition, some other variables that we have tested are also generating results that are worth mentioning, though their statistical significance levels are not below 0.05 to be reported in Fig. 2. They are subjective variables. Two are individual-specific immediate neighbourhood (level-1) factors, that is, the subjective sense of community (coefficient = − 0.02) and subjective perception of poor neighbourhood air quality (coefficient = 0.01),and two are wider shared neighbourhood (level-2) factors, that is, having companions to walk together (coefficient = − 0.89), and getting along well with neighbours (coefficient = − 0.30). We believe that the limited sample size may be a contributory factor and these subjective neighbourhood factors are worth closer examination in future larger-scale research.

## Discussion

Referring to the integrative framework of Billings and Moos [4], our work indicates that personal resources, such as physical health and body weight, are closely linked to the elderly’s mental health. This is contrary to previous studies which have showed that there is either no apparent association observed between BMI and depressive symptoms [27], or that there is even a negative relationship [30]. This present study suggests that maintaining a healthy weight allows one to be in better physical health condition and, in turn, has better mental health. Fitness programmes for the elderly, such as outdoor walking activities, could be further promoted at a community-wide level, in order to keep them active and physically fit.

In terms of environmental resources, we found that having larger areas of parks near one’s home is linked to fewer depressive symptoms. In Hong Kong and Singapore, parks represent open green space and leisure facilities such as playground, basketball courts and sports complex. One can conceptualize the impact in two ways. First, parks has been found to be important to people whether or not they use it often and actively [36]. Parks seen at a distance from one’s home can also be viewed as an environmental resource of importance to public mental health [1]. Second, being able to be directly immersed in the leisure environment provides opportunities for morning exercises, after-dinner walks, and social contacts, which are beneficial to older people’s health and well-being. Particularly in high-rise compact urban areas such as Hong Kong and Singapore, daily contact with the natural environment can have measurable mental benefits [10]. An earlier study indicated that once access is considered, size is more important than attractiveness in determining use [15]. This echoes the study result where the size of parks near to one’s home matters.

Another significant and powerful environmental resource that helps to reduce the risk of geriatric depression in Hong Kong and Singapore is promoting walkability within one’s neighbourhood. Walking is a popular, inexpensive, and convenient activity for older adults that is vital to maintaining their physical and mental health [32, 45]. A supportive neighbourhood environment helps promote walking as a mode of active transport that can be incorporated into the daily routine of older people, which often involves making medical trips [31]. By adopting an objectively measured micro-scale walkability assessment to evaluate different dimensions of the walking environment, this study found that promoting a more walking-friendly neighbourhood can be a core component of the public health policy. The composite score suggests that pavement width, surface condition, availability of street seats, ease of finding assistance, pedestrian crossing width, and whether or not the overpasses/underpasses are equipped with lifts, are important contributing factors in a walkable environment that decreases the risks of geriatric depression. A previous study discovered that, in a Western context, a more walking-friendly neighborhood decreases the risk among the elderly of becoming depressed [2].

## Conclusion

This study offers an integrated analysis of neighbourhood effects on geriatric depression; and provides empirical evidence suggesting that an older person’s neighbourhood of residence contributes to geriatric mental health. A walkable environment with abundant parks are favourable environmental settings for older people in Hong Kong and Singapore. To date, knowledge is very limited regarding the variability of neighbourhood effects among Asian cities on a person’s mental health. By exploring the effects of neighbourhood environment on geriatric depression, the results have paved the way for further studies on inter-city or regional comparison of mental health in an ageing population globally. The present study also adds to the diversity of available population-based literature on geriatric depression in Asia. Theoretically, the study stresses that the concept of geographical neighbourhood that goes beyond the immediate nearby areas but also the wider community. The first set of neighbourhood factors, which we referred to as level-1 local factors, can be individual-specific. It reflects one’s perceptions of his/her neighbourhood. The second set of neighbourhood characteristics, referred to as level-2 local factors, is related to certain general or common conditions that people share in a local area. The advantages at the different levels that the assessment revealed can provide a more robust understanding of the issues, while also underlining the challenges that exist in defining neighbourhoods; which is rather a complex concept to define and measure.

This study is not without its limitations. First, the sampling strategy could be improved by trying to reach community-dwelling elderly directly. Given the difficulties of getting a better sampling frame, convenience sampling was used. Moreover, the sample size is limited. As SEM with multiple explanatory factors perform better with bigger samples [29], increasing the sample size will allow us to model more variables and further explore the complex relationships of subjective variables with depression. Furthermore, respondents in Hong Kong and Singapore are pooled for the analysis, differences among communities and between cities are not examined in this study. Further research should address variations at different spatial scales. Second, the research investigated the presence of clinically relevant levels of depressive symptoms using GDS rather than the clinical diagnosis of depression. However, GDS is one of the most commonly used depressive symptoms measures in geriatric studies and has been shown to have good reliability for clinical diagnosis. Third, the dependent variable for the analytical data analysis is binary in nature. This can lead to concerns over the prevalence and incidence of depressive symptoms, and reduce the predictive power of the model. Though the binary approach adopted is sufficient in addressing the manifold question at hand, it does place constraints on a better understanding of geriatric depression. Fourth, the cross-sectional design adopted poses a number of challenges. It is not possible to unequivocally determine the direction of causation using the present cross-sectional data. For instance, depression at any given time might be influenced by current neighbourhood features. On the other hand, it can also be the result of cumulative exposure to neighbourhood features over many years. The effects of the neighbourhood environment on the development of depression is likely to have a time lag. This calls for further, preferably longitudinal, research to adequately explain the causal pathways by which a neighbourhood might affect health. Very often, depression is best viewed across a longitudinal time series which transects through the invasion history of depression into a person’s mind [39]. Besides, as our research did not collect the elderly residential history and decision-making information, we cannot conduct meaningful analysis about the residential self-selection issue. We recognize that focused research may further explore its relationship with depression.

On the way forward, the findings of this research have political implications for both Hong Kong and Singapore, as they are faced with rapidly ageing populations and socio-cultural changes. Overall, the high prevalence rates of geriatric depression in many ageing societies has led to public concerns. So far, the ageing policy has put much attention on the financial, healthcare and housing aspects. Nonetheless, this paper finds that the geographical neighbourhood in which an older person lives has a significant impact on his/her mental health, even after accounting for individual-level determinants. Even though depression is an individually experienced phenomenon, external factors including the neighbourhood environment are linked to its development. The development, evaluation, and dissemination of a new generation of programmes and structural interventions, especially on how to improve the neighbourhood environment, can be targeted towards improving the mental health of this population. Above all, understanding depression requires an analysis of the complex web of variables that gave rise to these specific individual circumstances. The expanding body of research on the effect of the neighbourhood environment on depression holds great promise, not only for achieving a more complete aetiological picture of the conditions, but also for delineating ways to understand and promote health and well-being in an ageing population. The findings of this study suggest that psychological health and the environment require greater age-specific policies if the elderly are to age well and successfully with good quality of life and life satisfaction.

## Data Availability

The datasets collected in the current study are available from the corresponding author on reasonable requests.
